# Independent Risk Factors for RBC Transfusion in Children Undergoing Surgery. Analysis of 14,248 Cases at a German University Hospital

**DOI:** 10.3390/children8080634

**Published:** 2021-07-25

**Authors:** Florian Piekarski, Vanessa Neef, Patrick Meybohm, Udo Rolle, Wilfried Schneider, Kai Zacharowski, Elke Schmitt

**Affiliations:** 1 Department of Anaesthesiology, Intensive Care Medicine and Pain Therapy, University Hospital Frankfurt, Goethe University Frankfurt, 60590 Frankfurt, Germany; Vanessa.Neef@kgu.de (V.N.); Kai.Zacharowski@kgu.de (K.Z.); Elke.Schmitt@kgu.de (E.S.); 2 Department of Anaesthesia, Intensive Care, Emergency and Pain Medicine, University Hospital Wuerzburg, 97080 Wuerzburg, Germany; Meybohm_P@ukw.de; 3 Department of Paediatric Surgery and Paediatric Urology, University Hospital Frankfurt, Goethe University Frankfurt, 60590 Frankfurt, Germany; Udo.Rolle@kgu.de; 4 Department of Paediatric Intensive Care Medicine, Goethe University Frankfurt, 60590 Frankfurt, Germany; Wilfried.Schneider@kgu.de; 5 Institute of Biostatistics and Mathematical Modelling, Department of Medicine, Goethe University, 60590 Frankfurt, Germany

**Keywords:** transfusion, children, patient blood management, paediatric, anaemia

## Abstract

Background: paediatric patients are vulnerable to blood loss and even a small loss of blood can be associated with severe shock. In emergency situations, a red blood cell (RBC) transfusion may become unavoidable, although it is associated with various risks. The aim of this trial was to identify independent risk factors for perioperative RBC transfusion in children undergoing surgery. Methods: to identify independent risk factors for perioperative RBC transfusion in children undergoing surgery and to access RBC transfusion rates and in-hospital outcomes (e.g., length of stay, mortality, and typical postoperative complication rates), a monocentric, retrospective, and observational study was conducted. Descriptive, univariate, and multivariate analyses were performed. Results: between 1 January 2010 and 31 December 2019, data from *n* = 14,248 cases were identified at the centre. Analysis revealed an RBC transfusion rate of 10.1% (*n* = 1439) in the entire cohort. The independent predictors of RBC transfusion were the presence of preoperative anaemia (*p* < 0.001; OR = 15.10 with preoperative anaemia and OR = 2.40 without preoperative anaemia), younger age (*p* < 0.001; ORs between 0.14 and 0.28 for children older than 0 years), female gender (*p* = 0.036; OR = 1.19 compared to male gender), certain types of surgery (e.g., neuro surgery (*p* < 0.001; OR = 10.14), vascular surgery (*p* < 0.001; OR = 9.93), cardiac surgery (*p* < 0.001; OR = 4.79), gynaecology (*p* = 0.014; OR = 3.64), visceral surgery (*p* < 0.001; OR = 2.48), and the presence of postoperative complications (e.g., sepsis (*p* < 0.001; OR = 10.16), respiratory dysfunction (*p* < 0.001; OR = 7.56), cardiovascular dysfunction (*p* < 0.001; OR = 4.68), neurological dysfunction (*p* = 0.029; OR = 1.77), and renal dysfunction (*p* < 0.001; OR = 16.17)). Conclusion: preoperative anaemia, younger age, female gender, certain types of surgery, and postoperative complications are independent predictors for RBC transfusion in children undergoing surgery. Future prospective studies are urgently required to identify, in detail, the potential risk factors and impact of RBC transfusion in children.

## 1. Introduction

Bleeding in children undergoing surgery, and the requirement for blood product transfusions, is a complex situation due to differences in blood volume and haemoglobin concentrations that are dependent on age and weight [1]. In general, paediatric patients are more vulnerable to blood loss because even a small loss can be associated with severe shock [1]. Further, particularly in children, transfusion of allogeneic blood products can be associated with various risks [2,3,4]. The risk of an adverse event after allogeneic blood transfusion is, in comparison to adults, 1.3 times higher in children over 1 year of age and 2.8 times higher in neonates [3,5]. Statistically, higher rates of allergic reactions, febrile non-haemolytic reactions, and acute haemolytic reactions are found more often in children than in adults [6]. Thus, a comprehensive risk-benefit assessment is required for any transfusion decision in children. Hence, in recent years, careful handling of perioperative blood products has become increasingly important. For the management of preoperative anaemia, restrictive transfusion strategies, and blood sparing techniques, a patient blood management (PBM) programme has evolved over the last decades [7,8]. Blood sparing techniques have always been the focus for paediatricians; however, studies dealing with this topic are rare. Although decreased transfusion rates for children in general and for those severely injured have been demonstrated, results for children undergoing surgery are scarce [9,10]. Currently, there are only a small number of studies in paediatric surgery that investigated transfusion strategies, such as from paediatric cardiac surgical patients [11].

Therefore, the goal of this trial was to identify independent risk factors for perioperative RBC transfusion in children undergoing surgery.

## 2. Materials and Methods

This study was approved by the Ethics Committee of the University Hospital Frankfurt, Goethe University (Ref: 19-338) and performed in accordance with the Declaration of Helsinki. The research was registered on ClinicalTrials.gov (NCT04349813) prior to data extraction and analysis.

### 2.1. Aim 

The primary aim was to identify independent risk factors for perioperative RBC transfusion in children undergoing surgery. Secondary aims were the assessment of RBC transfusion rates and in-hospital outcomes (e.g., length of hospital stay, mortality, and typical postoperative complication rates).

### 2.2. Data Acquisition 

In the German coding and billing system ‘Diagnosis-Related Groups (DRGs)’, surgeries and procedures are coded with a surgical or interventional procedural code (OPS), and diagnoses are coded with an international classification of disease (ICD) code, as reported previously [8,9]. 

Demographic (age and gender) and additional routine data (admission and discharge dates, discharge type, Hb value at admission and discharge, OPS and ICD codes, RBC, platelet, fresh frozen plasma (FFP), fibrinogen, and prothrombin complex concentrate (PCC) total consumption) from the hospital information system (ORBIS^®^, Agfa HealthCare, Bonn, Germany) were extracted, with help from the IT department, and consecutively validated by the statistician in charge.

### 2.3. Patient Inclusion and Exclusion Criteria

**Figure 1:**
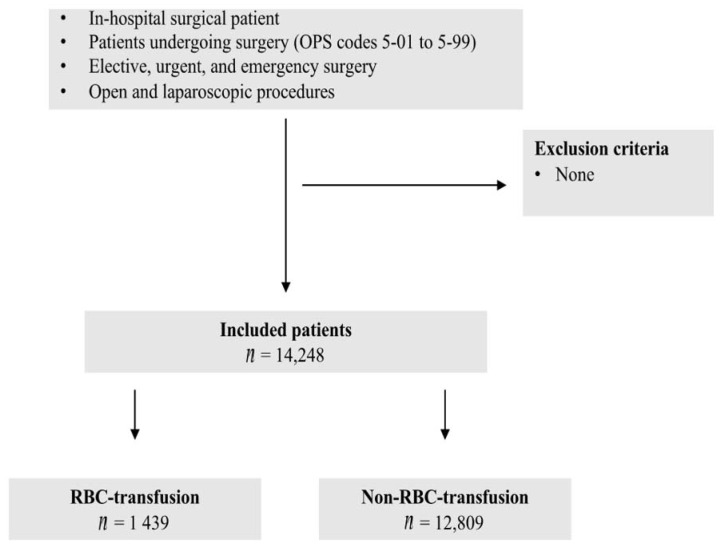
Flowchart of patient selection process.

All non-adult (under 18 years of age) in-hospital patients with a defined gender (female or male) who underwent elective, urgent, and emergency surgery (OPS codes 5-01 to 5-99) between 1 January 2010 and 31 December 2019 at the University Hospital Frankfurt were included in the analysis (Figure 1). Open and laparoscopic procedures were included. No exclusion criteria were defined.

### 2.4. Statistics

The study was monocentric, retrospective, and observational. Different surgical specialities were assigned to surgery groups according to their specific OPS codes (Supplementary file Table S1). Information on complications was obtained from the ICD codes (Supplementary file Table S2), information on in-hospital mortality was acquired through the discharge codes, and information on hospital length of stay was obtained via the admission and discharge dates. Anaemia was defined by the Hb values, according to the definition of the World Health Organization (WHO): 12–59 months of age lower than 11.0 g/dL, 5–11 years of age: <11.5 g/dL, 12–14 years: <12.0 g/dL, female 15–17 years of age: <12.0 g/dL, and male 15–17 years of age <13.0 g/dL [12].

Results of the descriptive analysis of patient characteristics (age and gender), anaemia and clinical outcomes (length of stay, mortality, and postoperative complications) are presented as means (±standard error), medians (with IQR), and rates (with 95% CI). Univariate analysis (Chi-square tests for binary endpoints and Wilcoxon-Mann-Whitney tests for continuous endpoints) were used to evaluate the influence of individual factors (e.g., age, type of surgery, and preoperative anaemia) on several outcomes (e.g., mortality, length of stay, and complications). Finally, multivariate regression analysis was performed to evaluate potential independent influence factors on RBC transfusion rate and other typical outcomes, and the results related *p*-values and odds ratios (ORs) are shown. All analyses were performed using the software R (version 3.6.3) [13].

## 3. Results

### 3.1. Patients’ Characteristics

Between 1 January 2010 and 31 December 2019, data from *n* = 14,248 cases were identified at the centre. Overall, there were *n* = 8651 (60.7%) male and *n* = 5597 (39.3%) female children. The mean age was 6.3 (± 0.0) years. The detailed demographic data are depicted in Table 1. 

### 3.2. RBC Transfusion

The analysis revealed an RBC transfusion rate of 10.1% (*n* = 1439) in the entire cohort. The median age was 2 years for transfused and 5 years for non-transfused children. A younger age was shown to be an independent predictor of RBC transfusion in the multivariate analysis, revealing that, for age groups older than 0 years of age, a significantly lower ratio of RBC transfused patients was predicted (*p* < 0.001 for all age groups, 1–4: OR = 0.28, 4–9: OR = 0.21, 10–14: OR = 0.18, and 15–17: OR = 0.14) (Table 2). RBC transfusion rates were the highest in the group of 0-year-old children (19.9%), whereas for the older children, the RBC transfusion rates were lower and comparable among each other (1–4: 7.4%, 4–9: 7.3%, 10–14: 8.8%, and 15–17: 7.7%).

Male children accounted for 55.9% of the transfused and for 61.3% of the non-transfused children. Descriptive analysis showed an RBC transfusion rate of 11.3% for the female gender and 9.3% for the male gender. Female gender was shown to be an independent predictor of RBC transfusion in the multivariate analysis (*p* = 0.036, OR = 1.19) (Table 2). 

The type of surgery was shown to be an independent predictor for RBC transfusion in the multivariate analysis, comparing the influence of each surgery group with the influence of the reference group of dermatology and ophthalmology: neurosurgery (*p* < 0.001; OR = 10.14), vascular surgery (*p* < 0.001; OR = 9.93), cardiac surgery (*p* < 0.001; OR = 4.79), gynaecology (*p* = 0.014; OR = 3.64), and visceral surgery (*p* < 0.001; OR = 2.48) (Table 2). Transfusion rates were highest for children who underwent cardiac (39.7%), neurosurgical (26.4%), and vascular (25.5%) surgeries. 

**Figure 2:**
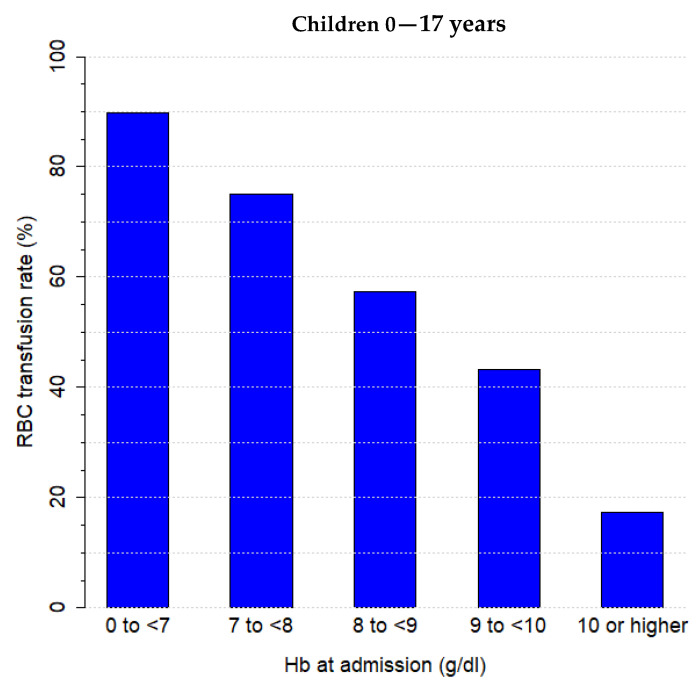
Proportion of patients transfused according to haemoglobin threshold. RBC transfusion rate (%) dependence on Hb value at admission (g/dL).

The preoperative anaemia rate was 82.4% in transfused children compared to an anaemia rate of 29.6% in non-transfused children (Table 3). Multivariate analysis revealed that preoperative anaemia was an independent predictor of RBC transfusion in children with preoperative anaemia (*p* < 0.001; OR = 15.10) and no preoperative anaemia (*p* < 0.001; OR = 2.40) (Table 2). Consequently, in the presence of preoperative anaemia, the transfusion rate was predicted to be approximately six-fold higher than without preoperative anaemia (Table 3). RBC transfusion rates were higher in the presence of preoperative anaemia as compared to the groups without (40.7% vs. 5.8%) and with unknown preoperative anaemia status (6.1%). As an additional analysis, RBC transfusion rates and corresponding preoperative Hb-values are shown in Figure 2. The results revealed that the lower the Hb value at admission, the higher the RBC transfusion rate.

Children who were transfused with RBC had a higher mortality rate (7.1% versus 0.1%) and a longer hospital LOS (median 32 versus 2 days, mean 42.0 vs. 4.4 days) than non-transfused children. 

Complications occurred more frequently in children who were transfused than in children who were not transfused: sepsis/systemic inflammatory response syndrome (SIRS) (23.6 vs. 0.4%), respiratory (41.2% vs. 2.7%), cardiovascular (24.5% vs. 1.0%), neurological (4.8% vs. 0.5%), renal (11.7% vs. 0.1%), and hepatic dysfunction (0.6% vs. 0.0%), respectively (Table 4). Multivariate analysis demonstrated that sepsis/SIRS (*p* < 0.001; OR = 10.16), respiratory (*p* < 0.001; OR = 7.56), cardiovascular (*p* < 0.001; OR = 4.68), neurological (*p* = 0.029; OR = 1.77), and renal dysfunction (*p* < 0.001; OR = 16.17) were independent predictors for RBC transfusion (Table 2). RBC transfusion rates were higher in the presence of complications than without: sepsis/SIRS (88.3% vs. 7.9%), respiratory (63.4% vs. 6.4%), cardiovascular (72.8% vs. 7.9%), neurological (54.3% vs. 9.7%), renal (92.3% vs. 9.0%), and hepatic dysfunction (100.0% vs. 10.0%), respectively.

## 4. Discussion

In this study, we were able to show that 10.1% of children undergoing surgery required an RBC transfusion. Independent predictors of RBC transfusion were the presence of preoperative anaemia, younger age, female gender, certain types of surgery, sepsis, respiratory, cardiovascular, neurological, and renal dysfunction.

Preoperative anaemia is known to significantly impact RBC transfusion rate, mortality, and hospital LOS [14,15,16]. The results of our study also revealed, through multivariate analysis, that preoperative anaemia was an independent predictor of RBC transfusion, leading to approximately a six-fold higher transfusion rate. Thus, RBC transfusion rates were higher in the presence of preoperative anaemia than without (40.7% vs. 5.8%) and as compared to the group with unknown preoperative anaemia status (6.1%). This difference is, therefore, more evident than in the study of Faraoni et al., who investigated the influence of anaemia on the postoperative outcome in children undergoing surgery. The authors revealed that in children with and without anaemia, 12% and 11% were transfused, respectively [14]. In an analysis by Goobie et al., the influence of transfusion volumes on outcome was investigated [17]. In their multivariate analysis, age, gender, and type of surgery were found to be significant factors influencing RBC transfusion, as was also the case in our study [17]. Stey et al. revealed that young age (<2 years of age), high ASA classification IV, septic shock, and preoperative resuscitation were predictors for intra- and postoperative RBC transfusion [18]. Thus, as was found in our study, we were able to demonstrate younger age as a significant predictor for RBC transfusion.

What conclusions can be drawn from the results, and how can RBC transfusions be reduced to the absolute minimum? A central point here is the identification of anaemia and early therapy as a module of a PBM programme. Thus, the implementation of PBM programmes in paediatric institutions has increasingly been investigated [2,19,20,21]. Guidelines for the introduction of PBM programmes in children undergoing surgery are already available [1,22]. In addition to preoperative anaemia optimization, the question arises as to when a child should be transfused. It has to be clarified that there is limited evidence, rather than expert opinions [1], on the indication for transfusions in children as underlined by a recent review article [14]. However, guidelines do provide appropriate recommendations. The European Society of Anaesthesiology and Intensive Care (ESAIC) recommends a haemoglobin level in the range of 7.0 to 9.0 g/dL for bleeding children [23]. Liberal versus restrictive transfusion strategies in children have been compared in prospective randomized controlled trials in cardiac surgery and intensive care medicine [11,20]. The data indicate no differences in complications, clinical outcomes or mortality between the liberal (10.8 g/dL) and restrictive (8.0 g/dL) groups in children undergoing cardiac surgery [11]. A patient-individual indication for RBC transfusion is recommended. Individual transfusion triggers, such as pre-existing diseases and parameters for an insufficient oxygen supply or catecholamine requirement, should be considered. 

### Limitations

There are some study limitations. The analysis is retrospective by design and, therefore, the data are not completely tailored to all study aims. However, the data were queried and analysed according to standardized methods that were already used in other research projects conducted by our study group. On the basis of the database, no distinction could be made between emergency, urgent, or elective surgery. The data set did not allow for further differentiation of the surgery, surgical technique, and ASA status. Furthermore, in the context of PBM, unnecessary blood tests should be avoided and only performed in cases when indicated. Due to this issue, blood samples were not drawn in the majority of children prior to surgery. Hypothetically, children without preoperative blood sampling may be healthier and transfused in comparably low proportions compared to children without preoperative anaemia. However, a bias arises here: the exclusion of children without a known Hb value, as was implemented in other studies, may lead to a change in all other influencing parameters because only sicker children are supposedly included in the study. Prospective studies that include all required data in detail are, therefore, necessary to answer these questions. On the other hand, our study can provide useful indications to design future prospective studies and has the advantage of a high number of cases, which would not be feasible in prospective studies. 

## 5. Conclusions

The present study demonstrates that preoperative anaemia, younger age, female gender, certain types of surgery, and postoperative complications are predictors for RBC transfusion in children undergoing surgery. Future prospective studies are urgently required to identify the potential risk factors and impact of RBC transfusion in children. 

## Figures and Tables

**Table 1:** Patient characteristics and type of surgery.

	RBC-Transfusion *n* (%) 1439 (10.1%)	Non-RBC-Transfusion *n* (%) 12,809 (89.9%)	*p*-Value
**Patient characteristics**			
Gender male	55.9% (53.3–58.5%); *n* = 805	61.3% (60.4–62.1%); *n* = 7846	<0.001
Age (years)	4.9 (±0.1), 2.0 (0.0; 9.0)	6.4 (±0.0), 5.0 (1.0; 11.0)	<0.001
Proportion of Age groups			<0.001
0	38.6%; *n* = 555	17.5%; *n* = 2240	
1–4	21.3%; *n* = 307	29.9%; *n* = 3829	
5–9	15.3%; *n* = 220	21.8%; *n* = 2796	
10–14	15.0%; *n* = 216	17.5%; *n* = 2242	
15–17	9.8%; *n* = 141	13.3%; *n* = 1702	
**Type of surgery**			
Proportion of surgery groups			<0.001
Dermatology, Ophthalmology	3.4%; *n* = 49	12.2%; *n* = 1567	
Neurosurgery	10.0%; *n* = 144	3.1%; *n* = 401	
Otorhinolaryngology	1.3%; *n* = 19	32.0%; *n* = 4096	
Thoracic surgery	0.7%; *n* = 10	0.4%; *n* = 55	
Cardiac surgery	2.0%; *n* = 29	0.3%; *n* = 44	
Vascular surgery	16.5%; *n* = 238	5.4%; *n* = 697	
Visceral and endocrine surgery	18.7%; *n* = 269	12.7%; *n* = 1621	
Urology	1.0%; *n* = 15	6.2%; *n* = 796	
Gynaecology	0.3%; *n* = 5	0.7%; *n* = 84	
Obstetric	0.1%; *n* = 1	0.4%; *n* = 52	
Oral-maxillofacial surgery	0.0%; *n* = 0	1.4%; *n* = 178	
Trauma and orthopaedic surgery	1.3%; *n* = 18	11.0%; *n* = 1403	
Haematopoietic and lymphatic system	18.8%; *n* = 271	1.5%; *n* = 194	
Mixed group	25.8%; *n* = 371	12.7%; *n* = 1621	

**Table 2:** Predictors for RBC transfusion.

Risk Factor	Univariate Analysis	Multivariate Analysis
	*p*-Value	*p*-Value; OR (with 95% CI)
**Gender**	<0.001	*p* = 0.036; OR = 1.19 (1.01–1.39) (female compared to male)
**Age (years)** 0 1–4 5–9 10–14 15–17	<0.001	(all compared to age 0) *p* < 0.001; OR = 0.28 (0.20–0.38) *p* < 0.001; OR = 0.21 (0.15–0.30) *p* < 0.001; OR = 0.18 (0.13–0.26) *p* < 0.001; OR = 0.14 (0.10–0.21)
**Anaemia at admission** Anaemia No anaemia Unknown ^1^	<0.001	*p* < 0.001; OR = 15.10 (11.58–19.90) *p* < 0.001; OR = 2.40 (1.76–3.30) (all compared to unknown)
**Type of surgery** Dermatology, Ophthalmology Neurosurgery Otorhinolaryngology Thoracic surgery Cardiac surgery Vascular surgery Visceral and endocrine surgery Urology Gynaecology Obstetric Oral-maxillofacial surgery Trauma and orthopaedic surgery Haematopoietic and lymphatic system Mixed group	<0.001	(all compared to Derm. and Oph.) *p* < 0.001; OR = 10.14 (6.48–16.19) *p* < 0.001; OR = 0.29 (0.15–0.53) *p* = 0.598; OR = 1.31 (0.46–3.36) *p* < 0.001; OR = 4.79 (2.05–11.04) *p* < 0.001; OR = 9.93 (6.48–15.59) *p* < 0.001; OR = 2.48 (1.64–3.83) *p* = 0.676; OR = 1.17 (0.55–2.35) *p* = 0.014; OR = 3.64 (1.16–9.51) *p* = 0.559; OR = 0.55 (0.03–2.71) *p* = 0.963; OR = 0.00 (0.00–0.00) *p* = 0.669; OR = 1.15 (0.60–2.13) *p* < 0.001; OR = 36.98 (23.51–59.41) *p* < 0.001; OR = 5.86 (3.91–8.99)
Sepsis/SIRS ^2^	<0.001	*p* < 0.001; OR = 10.16 (6.98–15.07)
Respiratory dysfunction	<0.001	*p* < 0.001; OR = 7.56 (6.01–9.52)
Cardiovascular dysfunction	<0.001	*p* < 0.001; OR = 4.68 (3.43–6.40)
Neurological dysfunction	<0.001	*p* = 0.029, OR = 1.77 (1.06–2.97)
Renal dysfunction	<0.001	*p* < 0.001; OR = 16.17 (8.33–33.55)
Hepatic dysfunction	<0.001	*p* = 0.988; OR = 5.58 × 10^6^ (0.00–∞) ^3^

^1^ Blood sampling at admission is not a routine part of surgical care for children, unless it is necessary due to the disease severity. ^2^ Systemic inflammatory response syndrome. ^3^ Upper CI endless, due to non-transfusion for hepatic dysfunction (0.0% (0.0–0.0%); *n* = 0 non-transfused versus 0.6% (0.2–1.1%); *n* = 8 transfused.

**Table 3:** Haemoglobin values and transfusion rate.

Hb Values and Transfusion Rates	RBC-Transfusion *n* (%) 1439 (10.1%)	Non-RBC-Transfusion *n* (%) 12,809 (89.9%)	*p*-Value
Hb value at admission (g/dL)	10.9 (±0.1), 10.3 (8.4; 12.7)	12.6 (±0.0), 12.5 (11.1; 13.7)	<0.001
Anaemia rate at admission	82.4% (79.6–85.0%); *n* = 661	29.6% (28.0–31.2%); *n* = 963	<0.001
Proportion of anaemia groups at admission			<0.001
Anaemia at admission (%)	45.9%; *n* = 661	7.5%; *n* = 963	
No anaemia at admission (%)	9.8%; *n* = 141	17.9%; *n* = 2291	
Unknown anaemia status at admission ^1^	44.3%; *n* = 637	74.6%; *n* = 9555	
Hb value at discharge (g/dL)	10.1 (±0.1), 9.8 (8.7; 11.3)	12.3 (±0.0), 12.3 (11.2; 13.3)	<0.001
Anaemia rate at discharge	87.9% (85.4–90.1%); *n* = 711	25.6% (24.4–26.8%); *n* = 1393	<0.001
Proportion of anaemia groups at discharge			<0.001
Anaemia at discharge	49.4%; *n* = 711	10.9%; *n* = 1393	
No anaemia at discharge	6.8%; *n* = 98	31.6%; *n* = 4052	
Unknown anaemia status at discharge	43.8%; *n* = 630	57.5%; *n* = 7364	
Platelet transfusion rate	47.9% (45.3–50.6%); *n* = 690	0.7% (0.5–0.8%); *n* = 87	<0.001
FFP ^2^ transfusion rate	30.6% (28.2–33.0%); *n* = 440	0.4% (0.3–0.5%); *n* = 45	<0.001
Fibrinogen transfusion rate	3.3% (2.4–4.3%); *n* = 47	0.0% (0.0–0.1%); *n* = 5	<0.001
PCC ^3^ transfusion rate	3.3% (2.4–4.3%); *n* = 47	0.1% (0.1–0.2%); *n* = 19	<0.001

^1^ Blood sampling at admission is not a routine part of surgical care for children, unless it is necessary due to the disease severity. ^2^ FFP: fresh frozen plasma. ^3^ PCC: prothrombin complex concentrate.

**Table 4:** Complications.

Complications	RBC-Transfusion *n* (%) 1439 (10.1%)	Non-RBC-Transfusion *n* (%) 12,809 (89.9%)	*p*-Value
Sepsis/SIRS ^1^	23.6% (21.4–25.8%); *n* = 339	0.4% (0.3–0.5%); *n* = 45	<0.001
Respiratory dysfunction	41.2% (38.7–43.8%); *n* = 593	2.7% (2.4–3.0%); *n* = 342	<0.001
Cardiovascular dysfunction	24.5% (22.3–26.8%); *n* = 353	1.0% (0.9–1.2%); *n* = 132	<0.001
Neurological dysfunction	4.8% (3.7–6.0%); *n* = 69	0.5% (0.3–0.6%); *n* = 58	<0.001
Renal dysfunction	11.7% (10.1–13.4%); *n* = 168	0.1% (0.1–0.2%); *n* = 14	<0.001
Hepatic dysfunction	0.6% (0.2–1.1%); *n* = 8	0.0% (0.0–0.0%); *n* = 0	<0.001
Mortality	7.1% (5.8–8.5%); *n* = 102	0.1% (0.0–0.1%); *n* = 9	<0.001
Hospital LOS ^2^ (days)	42.0 (± 1.1), 32.0 (13.0; 53.0)	4.4 (± 0.1), 2.0 (1.0; 4.0)	<0.001

^1^ Systemic inflammatory response syndrome. ^2^ LOS: length of stay.

## Data Availability

The data that support the findings of this study are available from the corresponding author upon reasonable request.

## Supplementary Materials

The following are available online at https://www.mdpi.com/article/10.3390/children8080634/s1, Supplement I surgical specialties (OPS Codes) and Supplement II coding of the postoperative complications (ICD-Codes).
