# Supplementary material for: Independent Risk Factors for RBC Transfusion in Children Undergoing Surgery. Analysis of 14,248 Cases at a German University Hospital
Source: Children (Basel). 2021 Jul 25;8(8):634. doi: 10.3390/children8080634 (PMC8394932; doi:10.3390/children8080634)
Supplement: Supplementary file 1 [file children-08-00634-s001.zip › children-1309510-supplementary.pdf]

Supplement

Table S1. Surgical specialties (OPS Codes)

Number	Surgery groups	OPS codes
0	Dermatology, Ophthalmology, Unspecific	Only 5-08 to 5-16, 5-89 to 5-99
1	Neurosurgery	Only 5-01 to 5-05*
2	Otorhinolaryngology	Only 5-18 to 5-31*
3	Thoracic surgery	Only 5-32 to 5-34*
4	Cardiac surgery	Only 5-35 to 5-37*
5	Vascular surgery	Only 5-38 to 5-39*
6	Visceral and endocrine surgery	Only 5-06 to 5-07, 5-42 to 5-45*
7	Urology	Only 5-55 to 5-64*
8	Gynaecology	Only 5-65 to 5-71, 5-87 to 5-88*
9	Obstetric	Only 5-72 to 5-75*
10	Oral-maxillofacial surgery	Only 5-76 to 5-77*
11	Trauma and orthopaedic surgery	Only 5-78 to 5-86*
12	Operations on the haematopoietic and lymphatic system	Only 5-40 to 5-41*
13	Mixed	OPS from at least two different Surgery specialties 1–12*

* The surgery groups 1–13 may also contain in addition OPS from the surgery group 0.

Table S2. Coding of the postoperative complications (ICD-Codes)

Complications	ICD codes
Cardiovascular dysfunction	I21 I21.0 I21.1 I21.2 I21.3 I21.4 I21.9 I22 I22.0 I22.1 I22.8 I22.9 I46 I46.0 I46.1 I46.9 I95.2 I95.8 I95.9 R57.0 R57.1 R57.2 R57.9 T88.2 T81.1 T79.4 R00.0 R00.1 R00.3 P29.0 P29.1 P29.4 P29.8 P29.9
Neurological dysfunction	I63 I63.0 I63.1 I63.2 I63.3 I63.4 I63.5 I63.6 I63.8 I63.9 I64 G93.1 G93.4 G93.6 F05.0 F05.8 F05.9 R40 R40.0 R40.1 R40.2P91.0 P91.4 P91.5 P91.6 P91.8 P91.9
Renal dysfunction	N17 N17.0 N17.1 N17.2 N17.8 N17.9 N19 N99.0 R34
Respiratory dysfunction	J12 J12.0 J12.1 J12.2 J12.3 J12.8 J12.9 J13 J14 J15 J15.0 J15.1 J15.2 J15.3 J15.4 J15.5 J15.6 J15.7 J15.8 J15.9 J16 J16.0 J16.8 J18 J18.0 J18.1 J18.2 J18.8 J18.9 J96.0 J96.9 J95.1 J95.2 P28.5 J80 P22 P22.0 P22.1 P22.8 P22.9
Sepsis / SIRS	A40 A40.0 A40.1 A40.2 A40.3 A40.8 A40.9 A41 A41.0 A41.1 A41.2 A41.3 A41.4 A41.51 A41.52 A41.8 A41.9 A42.7 B00.7 B37.7 R65 R65.0 R65.1 R65.2 R65.3 R65.9 P36
Hepatic dysfunction	K72.0 K72.7 K72.9
